# Dreaming under anesthesia: is it a real possiblity? Investigation of the effect of preoperative imagination on the quality of postoperative dream recalls

**DOI:** 10.1186/s12871-016-0214-1

**Published:** 2016-08-02

**Authors:** Judit Gyulaházi, Pál Redl, Zsolt Karányi, Katalin Varga, Béla Fülesdi

**Affiliations:** 1 Faculty of Medicine, Department of Anesthesiology and Intensive Care, University of Debrecen, Debrecen, Hungary; 2 Faculty of Dentistry, Department of Maxillofacial Surgery, University of Debrecen, Debrecen, Hungary; 3 Faculty of Medicine, Department of Internal Medicine, University of Debrecen, Debrecen, Hungary; 4 Department of Affective Psychology, Institute of Psychology, Eötvös Lóránd University, Budapest, Hungary

**Keywords:** Anesthesia, Dreaming, Imagination, Suggestion

## Abstract

**Background:**

Images evoked immediately before the induction of anesthesia by means of suggestions may influence dreaming during anesthesia. This study is a retrospective re-evaluation of the original prospective randomized trial.

**Methods:**

Dream reports were studied in two groups. In group 1. dreams of patients who received suggestions, and in group 2, those of the control group of patients who did not. The incidence of dream reports and the characteristics and the theme of the reported dreams were compared among the groups.

**Results:**

In general, the control and the psychological intervention groups were different in terms of dreaming frequency, and non-recall dreaming. The incidence of dream reports was significantly higher in the suggestion group (82/190 at 10 min and 71/190 at 60 min respectively) than in the control group (16/80 at 10 min and 13/80 at 60 min, respectively; p_10_ = 0.001 and p_60_ = 0.002). There were no differences in the nature (thought- like or cinematic), quality (color or B&W) and the mood (positive vs. negative) of the recalled dreams. In general, the contents of the imaginary favorite place and the reported dream were identical in 73.2 %. Among the topics most successfully applied in the operating theater were loved ones (83.8 %), holiday (77.8 %) and sport (63.6 %).

**Conclusion:**

The results of the present study suggest that dreams during anesthesia are influenced by suggestions administered immediately preceding anesthesia.

**Trial registration:**

The study was registered in ClinicalTrials.gov. Identifier: Q1 NCT01839201, Date: 12 Apr. 2013.

## Background

The problem of consciousness does not appear as a central neuroscientific issue only; it has a fundamental importance for every clinician dealing with patients with an altered state of consciousness. When performing general anesthesia, a modified state or loss of consciousness is brought about under reliable, controllable and reversible conditions. The study of how anesthesia affects the brain may be important constituents of scientific research regarding consciousness [1]. Sleep is a natural form of the unconscious state. People who have been awakened from different stages of sleep can recall dreams. Dreaming under anesthesia: is it a real possibility? For years anesthetists believed that there was no dreaming during anesthesia, yet, a portion of patients reported dreams after recovery from anesthesia. Initially it was hypothesized that the patients who spoke of their dreams had been awake during a period of anesthesia. In this case dreams might show a relationship to external events; they are „dream-like” processes, unpleasant and undesirable side-effects that can sometimes lead to posttraumatic stress syndrome. However, Aceto’s and Leslie’s as well as our patients recalled dreams characterized by contents that were not operative events but similar to that of their habitual dreams with predominant positive emotions [2–5]. It has been proved that dream reports can be obtained even after properly-carried out anesthesia with an adequate depth. The perioperative period is characterized by a spontaneously altered state of consciousness of our patients due to their illness, the operation, and defenselessness. This is exactly why suggestive communication is an effective tool in our arsenal of perioperative adjunctive therapies even without formal hypnosis induction [6, 7]. Suggestions used immediately before the induction of general anesthesia help us in guiding our patients’ imagination. Patients imagine their favorite place as a dream plan of their own choice which is emotionally important and pleasant to them. Guided imagination impacted the patients dream recalls experienced under recovery of general anesthesia. In addition to the subjective experience the characteristics of the recovery state supported the likelihood of dreaming. The recovery of the patients who reported dreaming was often accompanied by emotional manifestations corresponding to the dream content (smiles, anger, crying), elements of behavior in line with the dream (embracing arms, a foot pressing down on the accelerator). In the first statistical analysis of our study we examined the effect of the psychological method and the hypnotic agents on the incidence of dreams. It has been demonstrated that dream recalls are more frequent in patients with preoperative suggestions applied before and during induction. Furthermore, formation of dreams and dream recalls were dependent on the anesthetic technique, especially propofol as an induction agent [8].

This examination aims to assess how the characteristics of the dream recalls were influenced by the guided imagination used immediately before the induction of anesthesia as compared to control patients.

Finally, our question is whether dreams under anesthesia are generated in a “bottom-up” or a “top-down” manner, namely, is dream generation more closely related to perception or to mental imagery and perhaps off-line memory consolidation?

## Methods

The investigations were carried out between 2009 and 2012 by the anesthesia team of the Department of Anesthesiology and Intensive Care at the Oral and Maxillofacial Surgery ward of the Faculty of Dentistry, University of Debrecen, in a prospective, randomized fashion.

### Grouping of the patients

Patients were randomly allocated into three groups according to the psychological method as follows:

- *In the control group* spontaneous dreams of patients were assessed under anesthesia without suggestions.
- *In the suggestion group* patients received suggestions evoking their images exclusively in the operating theatre at the time of induction [9].
- *In the “dreamfilm group”* the patients worked out a dreamfilm-plan using the favorite place technique one day prior to surgery. At induction, the series of images prepared by suggestions was evoked.

#### The details of the psychological interventions

The suggestion technique itself starts with a relaxation exercise, using suggestions promoting calm, deep breathing and muscle relaxation. The patient is not simply asked to remember an event, the aim is to produce a feeling that they are “virtually” in their favorite place. Meanwhile the patient is involved in the imagination process in a dialogue form. It lasted about 5 min.

In all three of the previously listed groups 3 further subgroups were formed based on the anesthesiological technique used: **Subgroup 1**: anesthetic induction with etomidate (0,15-0,3 mg/kg), maintenance with sevoflurane (1 MAC, low-flow technique), **Subgroup 2**: anesthetic induction with propofol (1,5–2,5 mg/kg), maintenance with sevoflurane (1 MAC, low-flow technique) and **Subgroup 3** (TIVA group): anesthetic induction with propofol (1,5–2,5 mg/kg), maintenance with propofol (8 –10 mg/kg/h). Envelope randomization for the anesthesiological technique was carried out in the operating theatre, immediately before induction. Anesthesia was managed to ensure that hypnotic depth measured by BIS monitoring was between 40 and 60 throughout the entire time elapsed between intubation and wound closure. Monitoring started at the time point before induction of anesthesia and ended after total recovery of the patient, when awake state and return of adequate communication were reached.

**Fig. 1:**
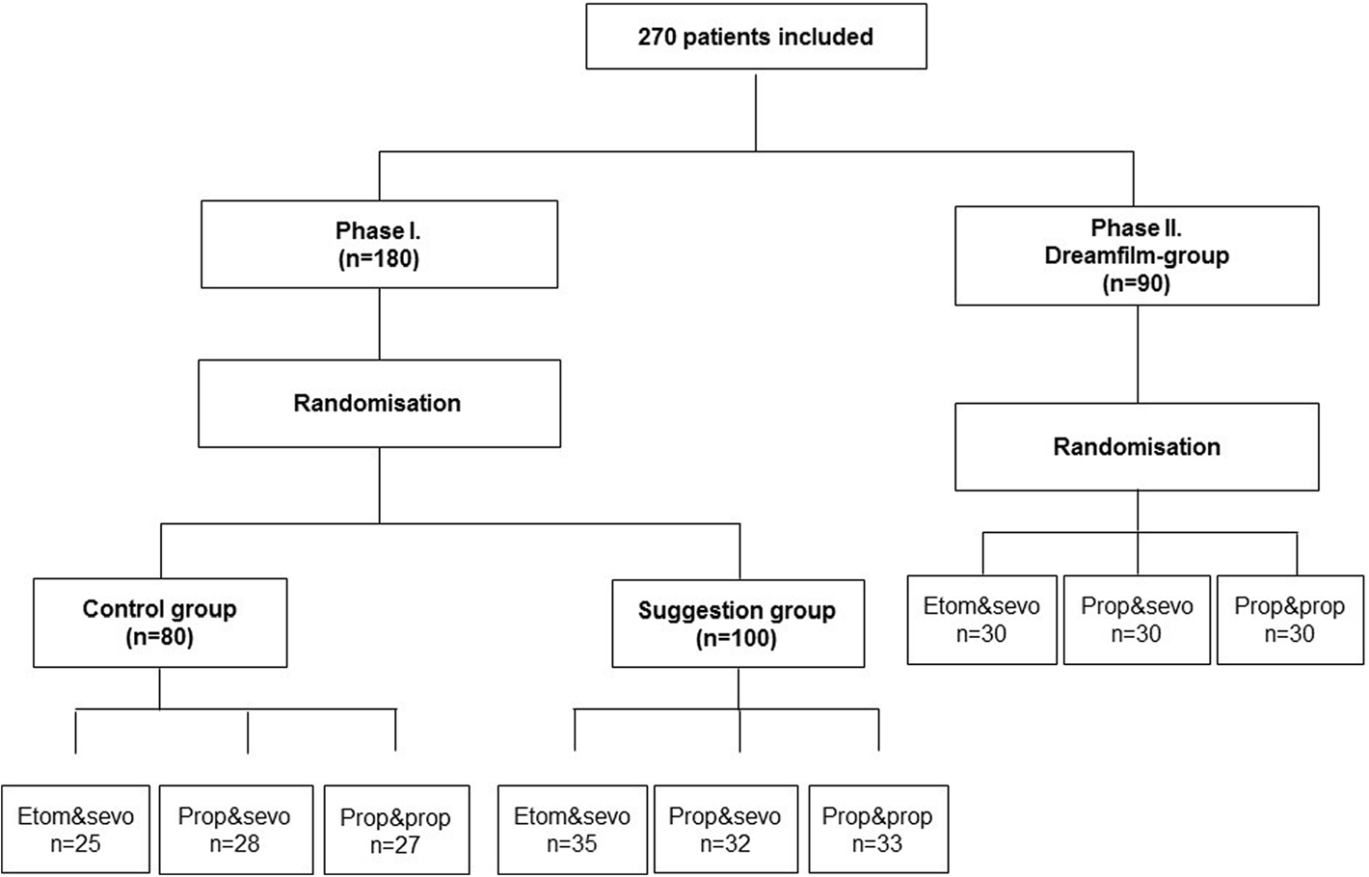
Inclusion of patients and randomisation

Figure 1 summarizes the inclusion of patients and randomization procedure [8].

In the present investigation we rearranged the data of our original research and subjected them to a new statistical analysis.

### Grouping of the dreams

Dreams were allocated into groups according to the following aspects:

1. *Dreams of the control group:* spontaneous dreams of patients were assessed under conventionally managed anesthesia without imaginations prior to surgery (all control groups; *n* = 80)
2. *Dreams of the suggestion group*: dreams of patients received suggestions evoking their images in the operating theater at the time of anesthesia induction (all suggestion and all dreamfilm groups of the original study: *n* = 190)

### Gathering data

The patients were interviewed about their dreams and the postoperative questionnaires were filled by the department’s assistants, 10 and 60 min after recovery (awake state of consciousness and return of adequate communication were reached). These were pretrained, independent (blind) staff personnel who were not present in the operating room and were not aware of the grouping status of the patients. The postoperative questionnaire contained parameters of the patients ’ general condition: blood pressure, pulse, complications, and communication. Questions about the dream report made up a pivotal part of this questionnaire. One section of the questionnaire concerned the assessment of the relationship between the anesthetist and the patient (rapport) as well as of the team’s work and the patient’s anxiety level related to the procedure.

The comparison of the imagination content and the dream content was conducted by two independent clinical psychologists who were unaware of the patients grouping after the research was completed (off-line analysis).

The key points of their evaluation were:

- Absence or presence of dream recalls within 10 and 60 min postoperatively (with recallable and non-recallable content).
- The characteristics of the recallable dreams:
  - nature: (thought or cinematic like)
  - quality: (color or black and white)
  - participant of the dream: (yes or no)
  - mood: (positive, negative)
  - content: (1: loved ones, 2: work 3: sports, 4: holidays, 5: erotic, 6: fairy tale, 7: religious, 8: other, 9: surgery)
  - connection to the imagination and dream content

### Statistical methods

The statistical analyses were performed with SAS for Windows 9.2 (SAS Institute Inc., SAS Campus Drive, Cary, North Carolina 27513.) statistical program package.

The variables were characterized by description analyses (case number, frequencies, mean and standard deviation) (proc. freq., proc univariate). The dream parameters between groups were compared by chi-square test (proc freq.).

The *p* <=0.05 probability values were accepted as significant.

## Results

A total of 270 patients were included in the study. Table 1 summarizes data on their confounding factors and preoperative history (data taken from [8]).

**Table 1:** Confounding factors and preoperative anamnestic data

		Control (*n* = 80)	Suggestion (*n* = 190)
Sex	female	51 (63.7 %)	118 (62.1 %)
	male	29 (36.3 %)	72 (37.9 %)
Age distribution	11–18 years	5 (6.3 %)	15 (7.9 %)
	19–30 year	25 (31.2 %)	66 (34.7 %)
	31–50 year	21 (26.2 %)	57 (30.0 %)
	51–75 years	29 (36.3 %)	50 (26.3 %)
	75 < yr	0 (0 %)	2 (1.1 %)
Frequency of dreaming per week at home	Mean (±SD)	2.69 (±2.19)	2.82 (±2.16)
Repeated dreams	yes	32 (40.0 %)	90 (47.4 %)
	none	48 (60.0 %)	100 (52.6 %)
Recalled home dreams	generally recalled	41 (51.2 %)	94 (49.4 %)
	sometimes recalled	26 (32.5 %)	63 (33.2 %)
	non-recalled	11 (13.8 %)	30 (15.8 %)
	no dreams at all	2 (2.5 %)	3 (1.6 %)
Present indication of surgery	accident	22 (27.5 %)	56 (29.5 %)
	cancer	19 (23.8 %)	51 (26.8 %)
	inflammatory	6 (7.5 %)	8 (4.2 %)
	reconstructive	10 (12.5 %)	25 (13.2 %)
	other	23 (28.8 %)	50 (26.3 %)
Level of preoperative anxiety	1 (weak)	4 (5.0 %)	6 (3.2 %)
	2	11 (13.8 %)	10 (5.3 %)
	3	28 (35.0 %)	68 (35.8 %)
	4	19 (23.7 %)	55 (28.9 %)
	5 (strong)	18 (22.5 %)	51 (26.8 %)
History of anesthesia	yes	54 (67.5 %)	104 (54.7 %)
	no	26 (32.5 %)	86 (45.3 %)
Experience by former anesthesia	neutral	24 (44.4 %)	49 (47.1 %)
	positive	19 (35.2 %)	40 (38.5 %)
	negative	11 (20.4 %)	15 (14.4 %)
Dream during former anesthesia	yes	1 (1.9 %)	11 (10.6 %)
	no	53 (98.1 %)	93 (89.4 %)
Recalled dream during former anesthesia	yes	1	7 (63.6 %)
	no	0	4 (37.4 %)

### Comparison of the dream recalls between the control and suggestions groups

(Table 2) revealed that in general, the control and the psychological intervention groups were different in terms of dreaming frequency, and non-recall dreaming. The incidence of dream reports was significantly higher in the suggestion group (82/190 at 10 min and 71/190 at 60 min respectively) than in the control group (16/80 at 10 min and 13/80 at 60 min, respectively; p _10_ = 0.001 and p_60_ = 0.002). Like in natural dreams, as time progressed, forgetting set in. Although there was a slight decrease in the incidence of dreams between 10 and 60 min both in the control and the suggestion groups (controls: from 16 to 13 vs. suggestion from 82 to 71), these differences did not reach the level of statistical significance (*p* = 0.54 for controls and *p* = 0.25 for suggestions, respectively). Similarly, the amount of non-dreamers did not change significantly between 10 and 60 min postoperatively in the control (10 min: 52/80 vs. 60 min: 57/80, *p* = 0.39) and in the suggestion group (10 min: 92/190 vs. 60 min: 96/190, *p* = 0.68).

**Table 2:** Number of dreamers, non-recall dreamers and non-dreamers in the control and psychological intervention groups at 10 and 60 min postoperatively

	Control (*n* = 80)	Suggestion & dreamfilm (*n* = 190)	*p*-value
	10 min postoperatively		
Dreamers	16	82	<0.01
Non-recall dreamers	12	16	
Non-dreamers	52	92	
	60 min postoperatively		
Dreamers	13	71	< 0.01
Non-recall dreamers	10	23	
Non dreamers	57	96	

### Assessment of dream features

The characteristics of the recallable dreams are summarized in Table 3. There were no differences in the nature (thought-like or cinematic), quality (color or B&W) and the mood (positive vs. negative) of the recalled dreams. In the control group characteristics of the dreams was cinematic in 82 %, and 76 % in the suggestion group (*p* = 0.62). In the control group the dreams were colorful in 87,5 % and black and white in 12,5 %, whereas they were colorful in 85,5 %, black and white 14,5 % in the suggestion group (*p* = 0.6). The mood of the dreams was dominantly good in both groups without any significant differences (87.5 % vs. 97.5 %; *p* = 0.06)**.**

**Table 3:** Characteristics of the recallable dreams

		Control group *n* = 16 dream recalls	Suggestion group *n* = 82 dream recalls	*p* value
Nature	thought like	0	15	0.06
	cinematic like	13	62	0.62
	didn’t remember	3	5	0.09
Quality	color	14	70	0.82
	black and white	2	12	0.82
Mood	positive	14	80	0.06
	negative	2	2	0.06

Notably, the patients were participants of their dreams in 100 % of the cases in the control group and in 97,7 % in the suggestion group.

### Analysis of dream topics

Comparison of the dream topics showed significant differences between the groups *p* < 0.001. The dreams that were reported contained elements of episodic memory (work, recreation, joint activities with loved ones) in both groups but imagination’s influencing effect could be observed (Table 4).

**Table 4:** Dream’s contents

	Control (*n* = 16)	Suggestion (*n* = 82)	*p*-value
Loved ones, family	3	45	<0.01
Work	6	9	<0.01
Sport	0	9	0.16
Holiday	1	15	0.23
Erotic	2	1	<0.05
Religious	0	2	0.52
Surgery	0	1	0.65
Other	4	0	<0.001

Chi-square test for multiple comparisons *p* value: <0.001

**Fig. 2:**
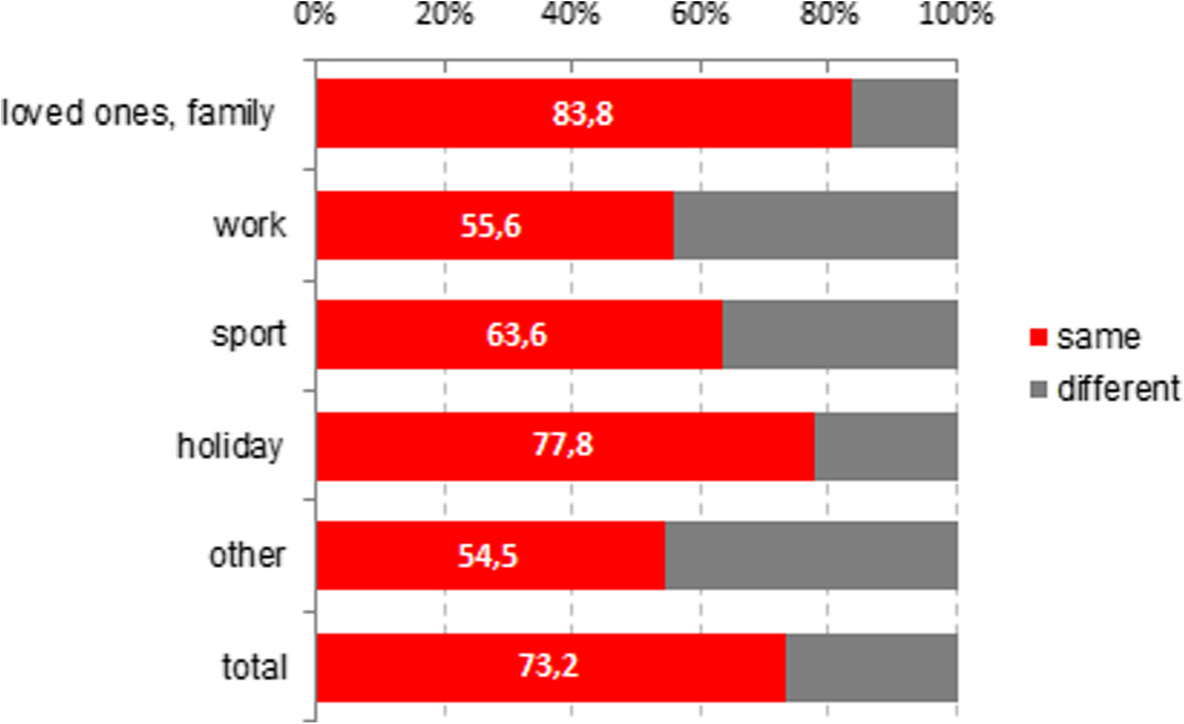
Agreement of the content of suggestion and dream recall in the suggestion group

In the final analysis we looked at how often the suggestions’ themes got manifested in the reported dreams. In general, the contents of the imaginary favorite place and the reported dream were identical in 73.2 %. Among the topics most successfully applied in the operating theater were loved ones (83.8 %), holiday (77.8 %) and sport (63.6 %) (Fig. 2).

## Discussion

In the present study we demonstrated that images evoked immediately before induction of anesthesia by means of suggestions may influence dreaming during anesthesia. Dream reports were obtained after properly-carried out anesthesia with an adequate depth.

The anesthetic state is very similar to NREM sleep. The patient is unconscious, processing of the external world stimuli is minimal and does not become conscious, either. The neural correlates of the two states show great similarities [10–19]. Thus it can be assumed that, via a similar mechanism, consolidation of episodic memory and dream formation may occur during anesthesia, too. The „Tetris phenomenon” described by Stickgold et al shed light on the relationship between experiences before falling asleep and NREM dream content. Studying the effect of practicing the Tetris game on NREM dreams, Stickgold et al found that it appeared in about 60 % of the subjects’ dreams during the next two nights. Sleep helped the consolidation of the memory traces of the Tetris game [20–22]. The phenomenon demonstrated in our study is similar to the Tetris phenomenon game at a phenomenological level. Like in the “Tetris phenomenon” the dream evoked with the help of suggestions before induction of anesthesia made its way into over 73,2 % of the dream content. The results of the present study suggest that dreams during anesthesia are probably the result of episodic memory consolidation of events immediately preceding anesthesia.

Dreams are characterized by reduced attention and voluntary control and volition, self-awareness and altered reflective thought, emotionality and altered mnemonic processes [23]. While REM dreams are widely known, the existence of NREM dreams was denied for a long time. In fact, between 40 and 60 % of patients emerging from NREM report dreams. NREM dreams are like thoughts, contain visual images, and rarely form a story or a scene. Subjects of investigations typically do not report dreams, rather, they report that they had thought of something or they had been somewhere. NREM dreams often contain elements of episodic memory, especially ones of the most recent, but sometimes older, life events, unlike REM dreams, where we typically find memory fragments, bizarre content that is difficult to tie to real events [24, 25]. Our experience with dreams during anesthesia is very similar to that described by Leslie [4, 5]. The dreams that were observed showed a great resemblance to NREM mentations (cinematic, colorful, with good mood) and contained elements of episodic memory (work, recreation, joint activities with loved ones). Dreams during anesthesia differ from REM dreams by a complete lack of bizarreness. The most obvious difference between dreaming and waking consciousness is the profound disconnection of the dreamer from his current environment. By definition a sleeping person shows no meaningful responses to external stimuli, unless they are strong enough to cause an awakening. It is known from previous reports that during deep sedation and anesthesia functional connections between the thalamus and the cortex–especially those mediating integration of cortical computations- are reduced, but connections mediating sensory transfer from the periphery are preserved [15, 26–28] As a result of this, the perception of the environmental stimuli is intact. This has been proven by evoked potential tests [29]. But patients are unconscious, because the cognitive binding after the perception of the external stimuli is the result of the activity and the intact connectivity of the frontoparietal (default mode) network. According to current knowledge, this is blocked during deep anesthesia [15, 17]. Moreover, stimuli not only fail to elicit a behavioral response, but also largely fail to be incorporated in the content of the dream. Just as dreams of sleep can incorporate contemporaneous sensory input (such as an alarm clock), near-miss awareness dreams under anesthesia may also incorporate sensory stimuli [4, 5]. Let us take an example from our personal experience to support this: During recovery the attending anesthetist asked the patient: ‘Can you hear me?’ As an answer, he recalled his dream by saying: ’I was at an excellent party, but a woman (the anesthetist) disturbed me in enjoying it, because she always asked after tiresome things’. This may suggest that during anesthesia, if hypnotic depth decreases temporarily, the events perceived by the patients may be incorporated into their ongoing dream in a new context. Examination of this phenomenon may be a future topic for further studies.

As a limitation of our study we have to mention that we did not address the issue of correlation between BIS and dream recall of the patient. Future studies aimed at investigating the factors influencing dreaming under anesthesia will undoubtedly provide valuable insight into the topic.

## Conclusions

Whenever the brain is disconnected from the environment under anesthesia, it can generate an entire world of conscious experiences by itself. Converging evidence supports the notion that dreaming under anesthesia is not generated by perception but rather may be closely related to imagination, and off-line episodic memory consolidation, where brain activity presumably flows in a “top-down” manner. Further studies are needed to prove this hypothesis. As this content can be manipulated by suggestions, this is a further tool to establish a positive emotional state during surgery that supposedly supports outcome and patient satisfaction.
